# Supplementary material for: Spin blockade and phonon bottleneck for hot electron relaxation observed in n-doped colloidal quantum dots
Source: Nat Commun. 2021 Jan 22;12:550. doi: 10.1038/s41467-020-20835-4 (PMC7822822; doi:10.1038/s41467-020-20835-4)
Supplement: Supplementary file 1 — Supplementary Information [file 41467_2020_20835_MOESM1_ESM.pdf]

Supplementary Information

Spin blockade and phonon bottleneck for hot electron relaxation observed in *n*-doped colloidal quantum dots

Wang et al.

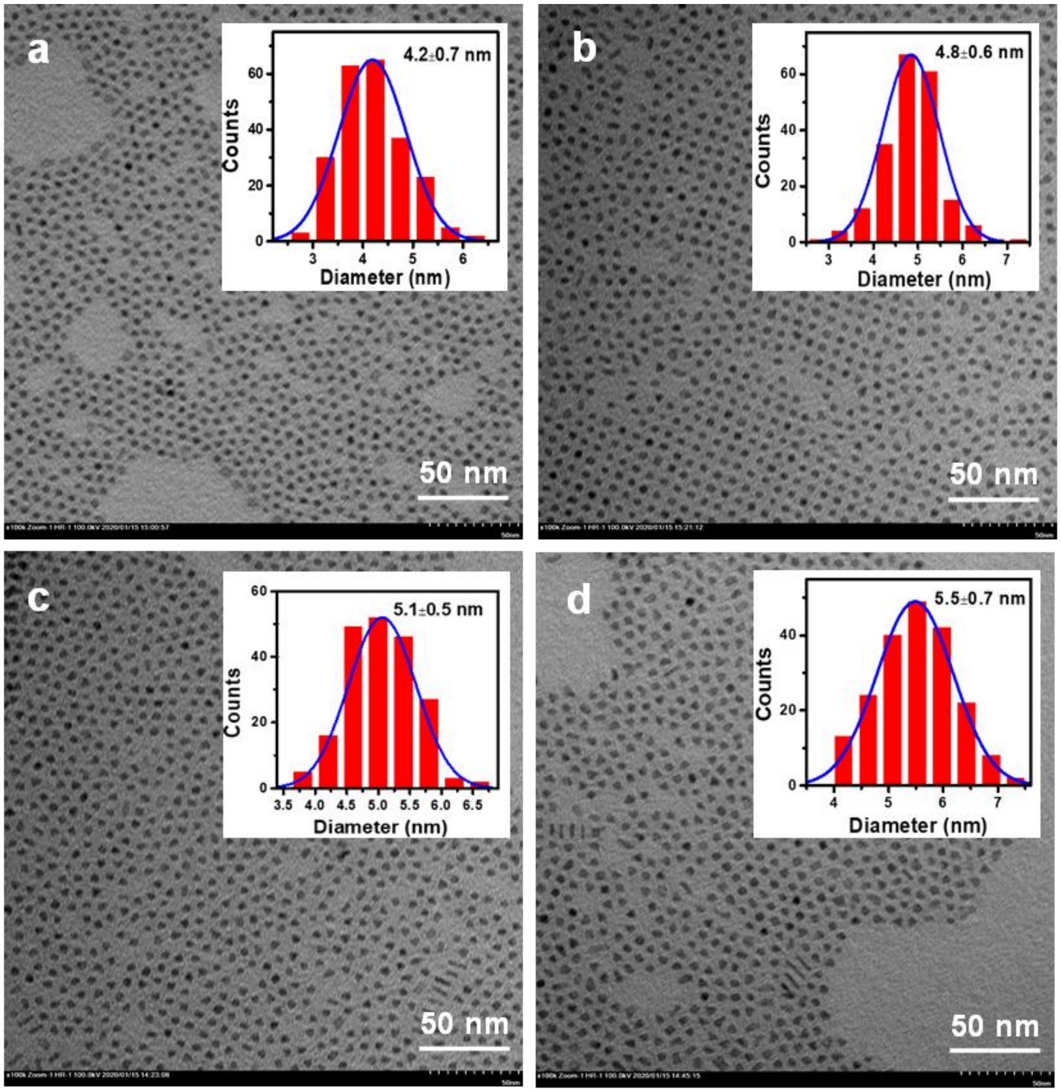

Supplementary Figure 1. QD morphologies. Transmission electron microscope (TEM) images of (a) 4.2 nm, (b) 4.8 nm, (c) 5.1 nm and (d) 5.5 nm CdSe QDs. Insets are their respective size distribution histograms.

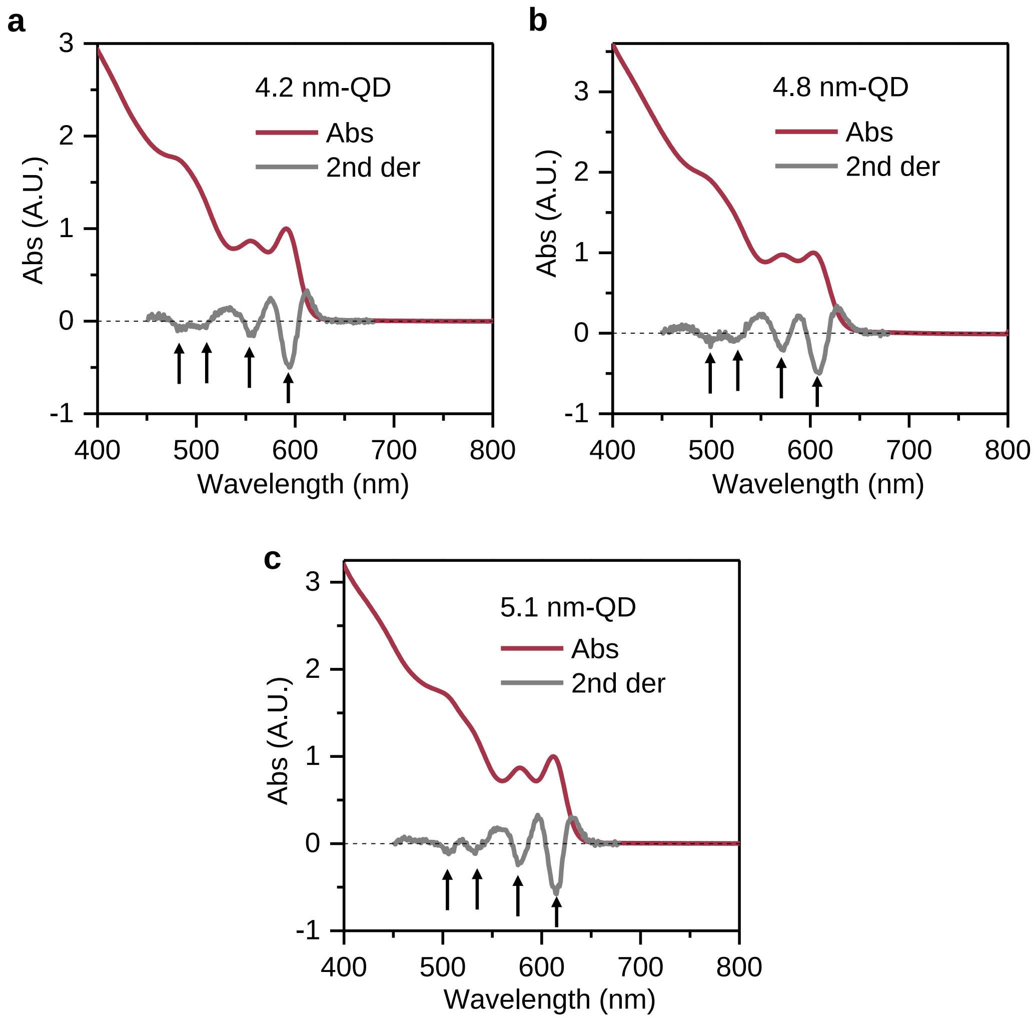

Supplementary Figure 2. Optical properties of QDs. Absorption spectra of (a) 4.2 nm, (b) 4.8 nm and (c) 5.1 nm CdSe QDs (red) and their second derivative spectra (grey).

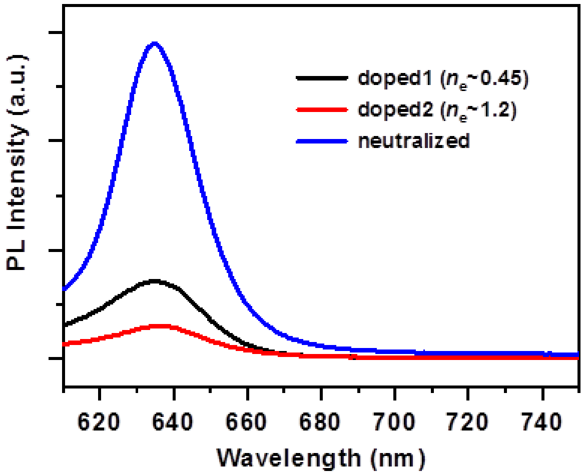

Supplementary Figure 3. PL of doped QDs. Photoluminescence (PL) spectra of two *n*-doped samples with nominal average numbers of band-edge electrons of 0.45 (black) and 1.2 (red), in comparison to that of the neutralized sample (blue).

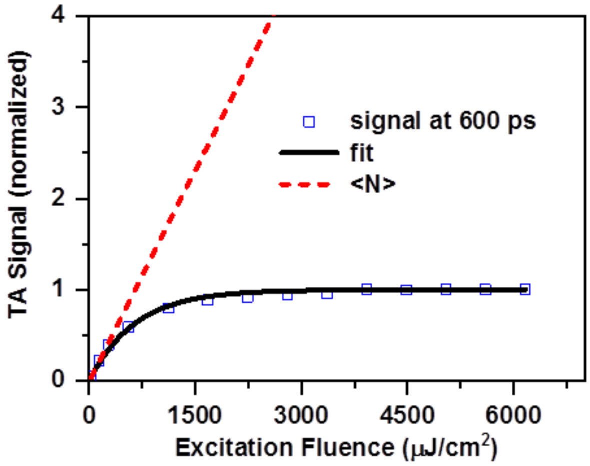

Supplementary Figure 4. Photons absorbed per QD. Scaled TA signal amplitude at 600 ps, after multiexciton Auger recombination has finished, as a function of the excitation fluence of the 547 nm pump (blue squares). The saturation curve is fitted to a Poissonian model for photon absorption (black solid line).[1] Specifically, the photon absorption statistics at a photon energy much higher than the bandgap is assumed to be a Poissonian, and hence after multiexciton Auger recombination the TA signal amplitude is simply proportional to the fraction of photoexcited QDs in the ensemble ($1-e^{-\langle N\rangle}$, $\langle N\rangle$ is the average number of photons absorbed per QD). $\langle N\rangle$ is proportional to the absorption cross section and excitation fluence. From the fit, we can determine $\langle N\rangle$ at any specific pump fluence (red dashed line). A typical pump fluence used in our experiment is 11.2 $\mu J/cm^2$, corresponding to $\langle N\rangle$ of ~0.017.

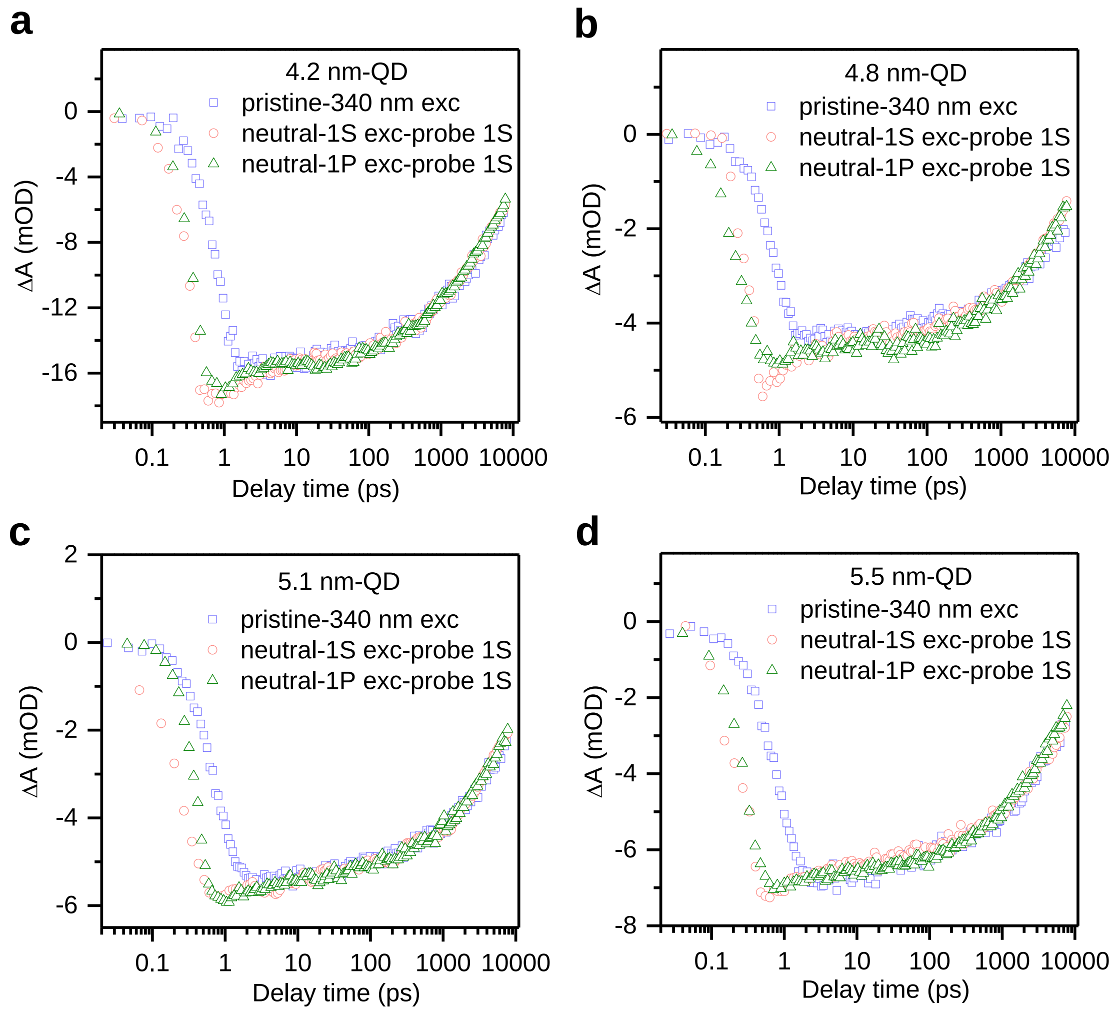

Supplementary Figure 5. Electron lifetime in QDs. 1S exciton bleach recovery kinetics of pristine QDs under 340 nm excitation (blue squares) and re-neutralized QDs under 1S (red circles) and 1P (green triangles) excitations for (a) 4.2 nm, (b) 4.8 nm, (c) 5.1 nm and (d) 5.5 nm CdSe QDs. The exciton bleach lifetime, which is a measure of the 1S electron lifetime, is barely affected by the doping and re-neutralization processes.

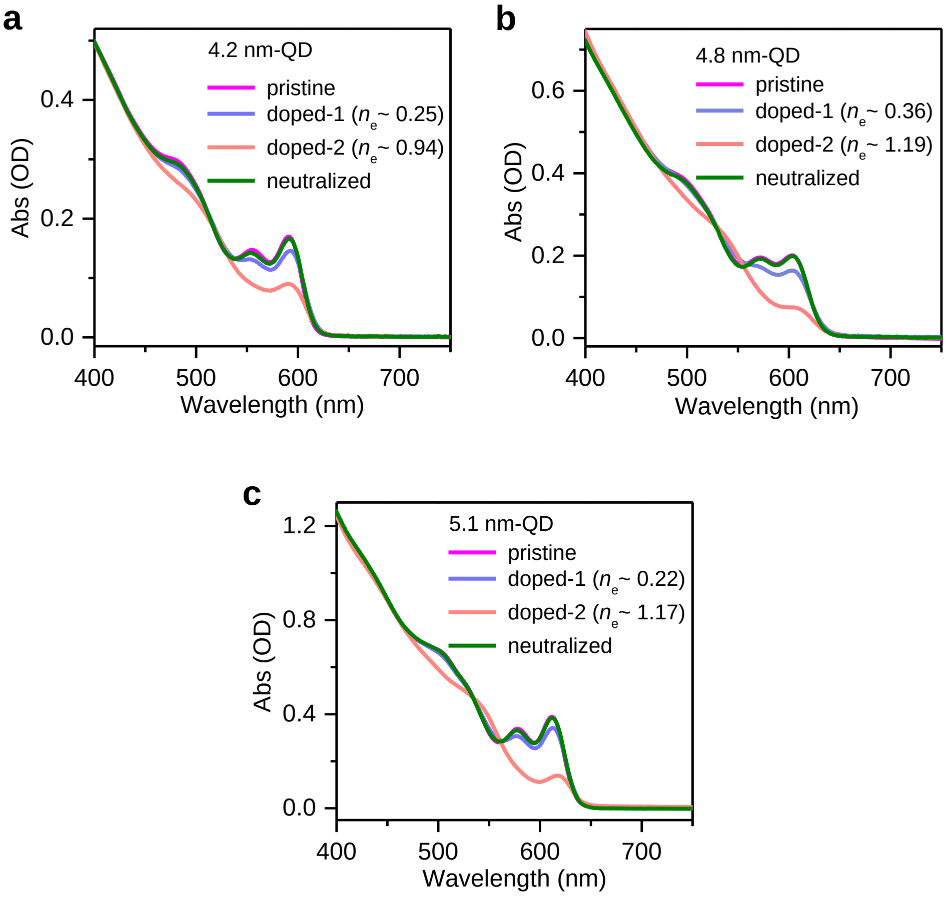

Supplementary Figure 6. Absorption spectra of *n*-doped QDs. Absorption spectra of the pristine (pink), *n*-doped-1 (blue), *n*-doped-2 (red) and re-neutralized (green) samples of (a) 4.2 nm, (b) 4.8 nm and (c) 5.1 nm CdSe QDs. The average numbers of doped conduction band electrons for *n*-doped-1 and *n*-doped-2 are estimated for each QD.

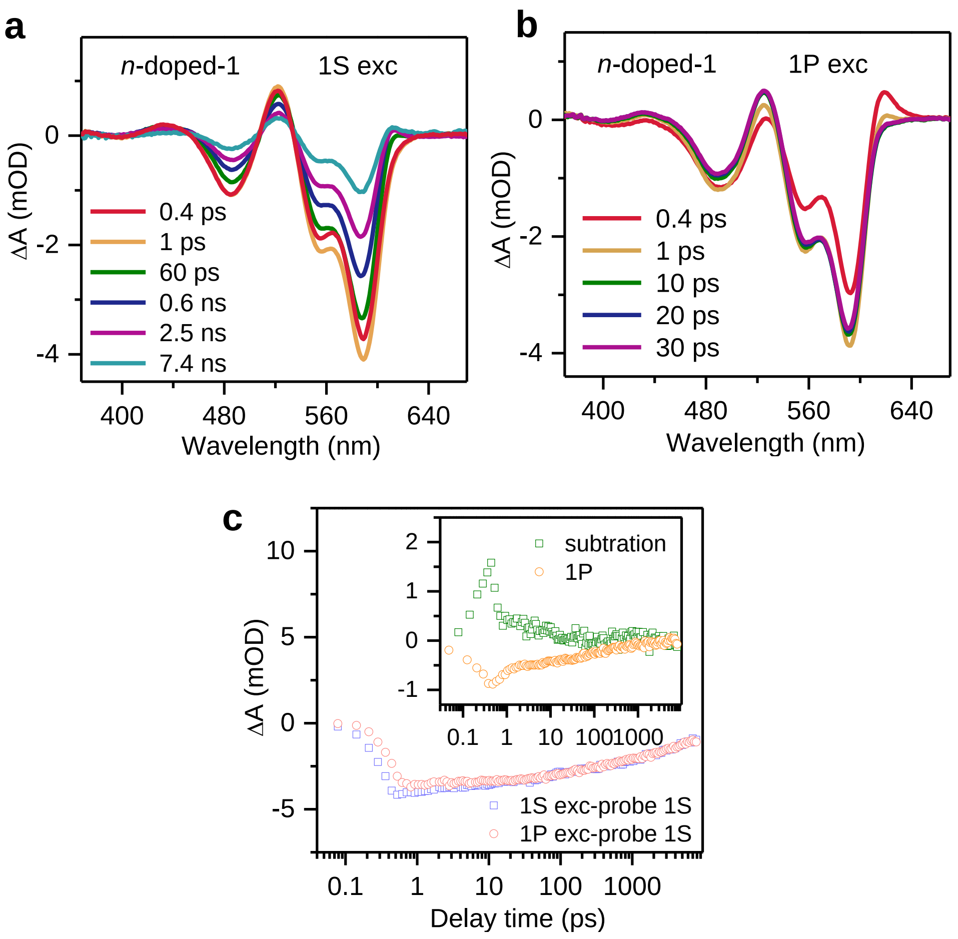

Supplementary Figure 7. Spin blockade in lightly *n*-doped 4.2 nm QDs. (a,b) TA spectra of *n*-doped-1 probed at indicated time delays following excitation at (a) $1S_e$-$1S_{3/2,h}$ and (b) $1P_e$-$1P_{3/2,h}$ transitions. (c) TA kinetics probed at the 1S bleach for *n*-doped-1 under $1S_e$-$1S_{3/2,h}$ (blue square) and $1P_e$-$1P_{3/2,h}$ (red circle) excitations, scaled at their slowly-decaying tail. Inset are the 1S kinetics obtained by performing a subtraction between the two traces in the main panel (green square) and the kinetics monitored at the 1P bleach (orange circle).

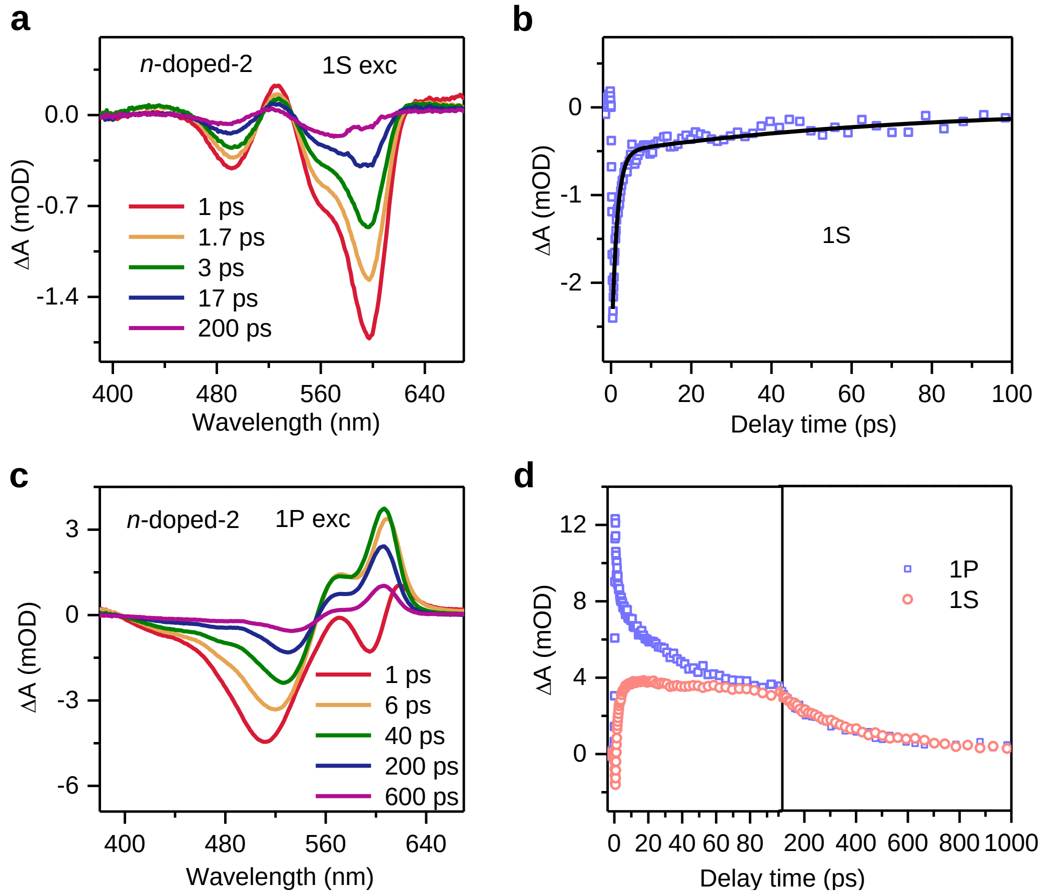

Supplementary Figure 8. Phonon bottleneck in heavily *n*-doped 4.2 nm QDs. (a,c) TA spectra of *n*-doped-2 probed at indicated time delays following excitations at (a) $1S_e$-$1S_{3/2,h}$ and (c) $1P_e$-$1P_{3/2,h}$ transitions. (b) TA kinetics probed at the 1S bleach for *n*-doped-2 under $1S_e$-$1S_{3/2,h}$ excitation (blue square). The black solid line is its bi-exponential fit to time constants of 1.4 (78%) and 74 ps (22%). (d) TA kinetics probed at the 1S absorption (red circle) and 1P bleach (blue square) for *n*-doped-2 under $1P_e$-$1P_{3/2,h}$ excitation.

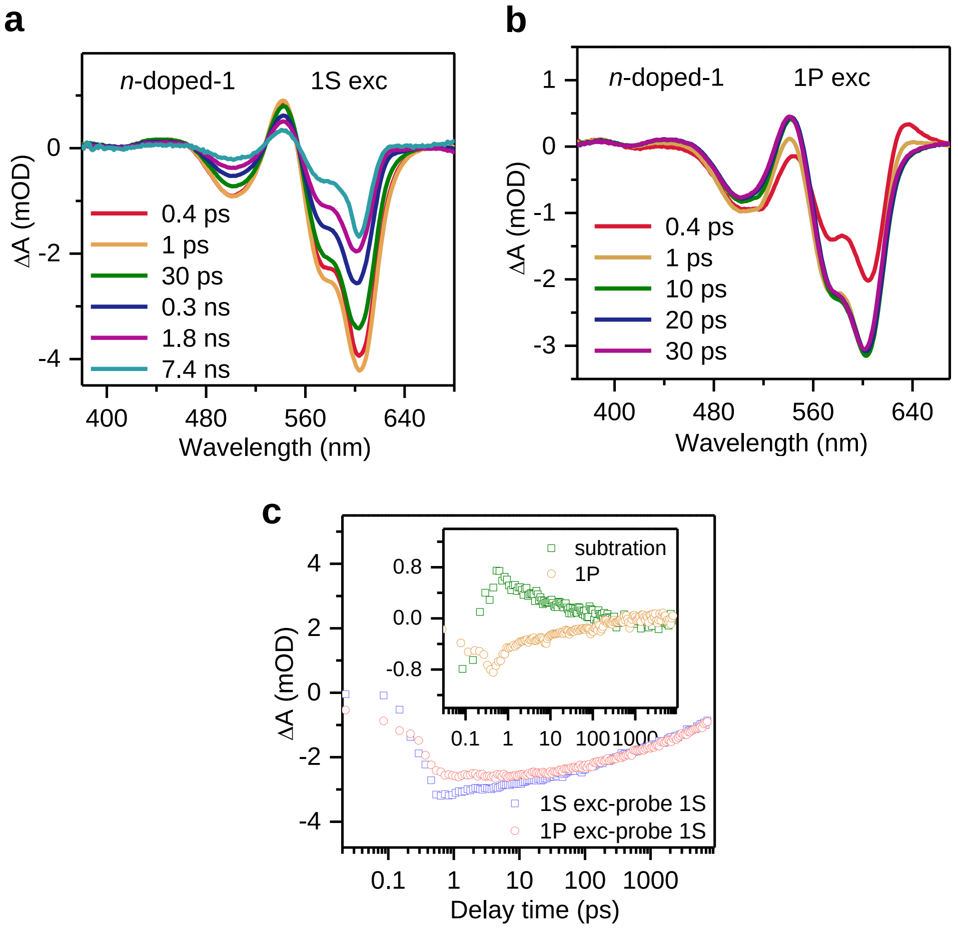

Supplementary Figure 9. Spin blockade in lightly *n*-doped 4.8 nm QDs. (a,b) TA spectra of *n*-doped-1 probed at indicated time delays following excitation at (a) $1S_e$-$1S_{3/2,h}$ and (b) $1P_e$-$1P_{3/2,h}$ transitions. (c) TA kinetics probed at the 1S bleach for *n*-doped-1 under $1S_e$-$1S_{3/2,h}$ (blue square) and $1P_e$-$1P_{3/2,h}$ (red circle) excitations, scaled at their slowly-decaying tail. Inset are the 1S kinetics obtained by performing a subtraction between the two traces in the main panel (green square) and the kinetics monitored at the 1P bleach (yellow circle).

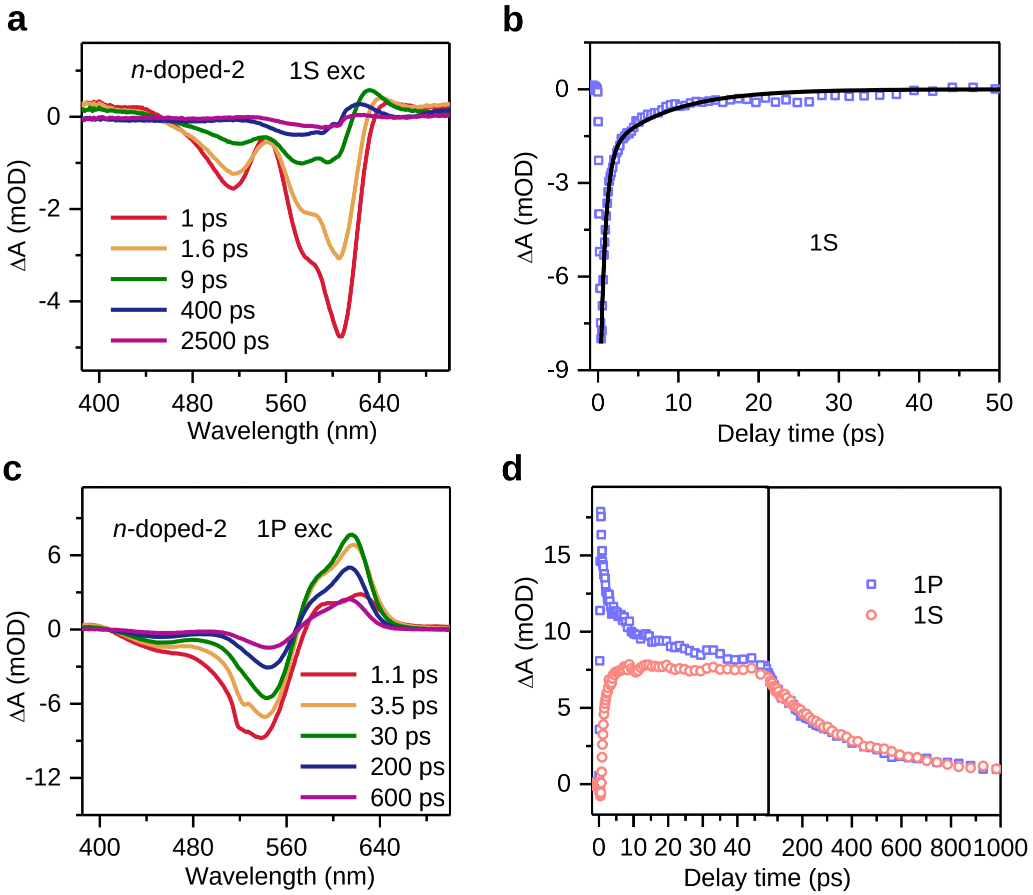

Supplementary Figure 10. Phonon bottleneck in heavily *n*-doped 4.8 nm QDs. (a,c) TA spectra of *n*-doped-2 probed at indicated time delays following excitations at (a) $1S_e$-$1S_{3/2,h}$ and (c) $1P_e$-$1P_{3/2,h}$ transitions. (b) TA kinetics probed at the 1S bleach for *n*-doped-2 under $1S_e$-$1S_{3/2,h}$ excitation (blue square). The black solid line is its bi-exponential fit to time constants of 0.6 (75%) and 7.7 ps (25%). (d) TA kinetics probed at the 1S absorption (red circle) and 1P bleach (blue square) for *n*-doped-2 under $1P_e$-$1P_{3/2,h}$ excitation.

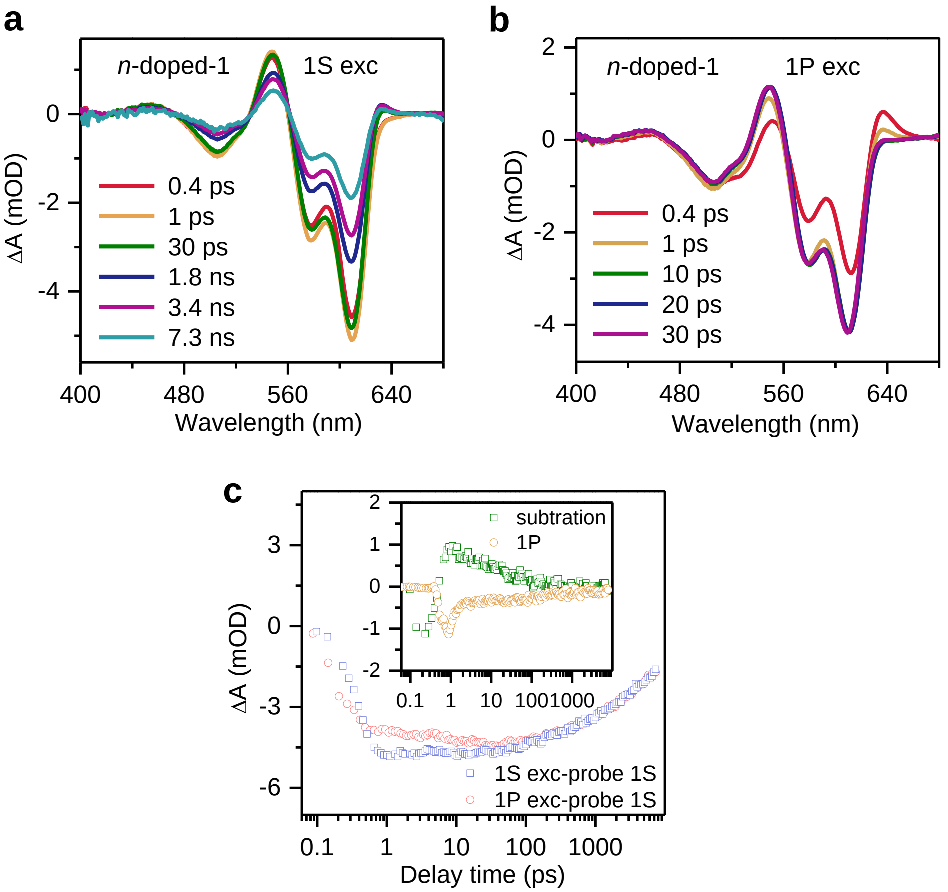

Supplementary Figure 11. Spin blockade in lightly *n*-doped 5.1 nm QDs. (a,b) TA spectra of *n*-doped-1 probed at indicated time delays following excitation at (a) $1S_e$-$1S_{3/2,h}$ and (b) $1P_e$-$1P_{3/2,h}$ transitions. (c) TA kinetics probed at the 1S bleach for *n*-doped-1 under $1S_e$-$1S_{3/2,h}$ (blue square) and $1P_e$-$1P_{3/2,h}$ (red circle) excitations, scaled at their slowly-decaying tail. Inset are the 1S kinetics obtained by performing a subtraction between the two traces in the main panel (green square) and the kinetics monitored at the 1P bleach (yellow circle).

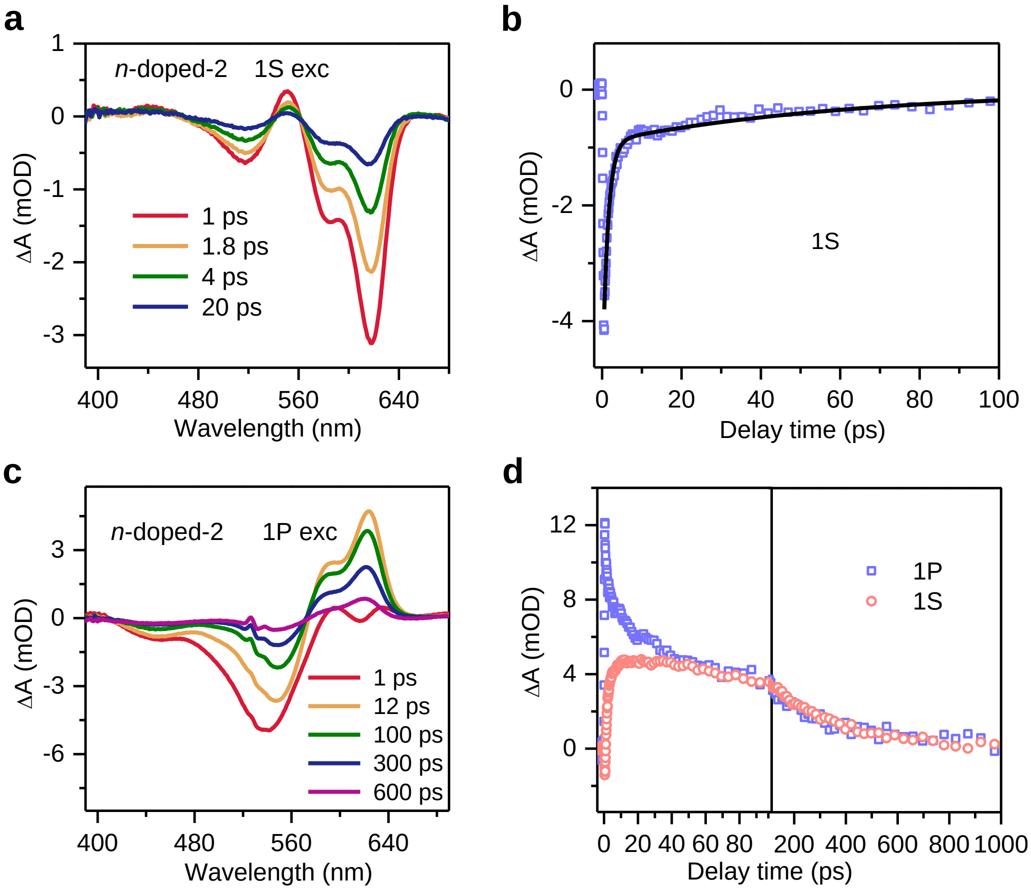

Supplementary Figure 12. Phonon bottleneck in heavily *n*-doped 5.1 nm QDs. (a,c) TA spectra of *n*-doped-2 probed at indicated time delays following excitations at (a) $1S_e$-$1S_{3/2,h}$ and (c) $1P_e$-$1P_{3/2,h}$ transitions. (b) TA kinetics probed at the 1S bleach for *n*-doped-2 under $1S_e$-$1S_{3/2,h}$ excitation (blue square). The black solid line is its bi-exponential fit to time constants of 1.4 (76%) and 63 ps (24%). (d) TA kinetics probed at the 1S absorption (red circle) and 1P bleach (blue square) for *n*-doped-2 under $1P_e$-$1P_{3/2,h}$ excitation.

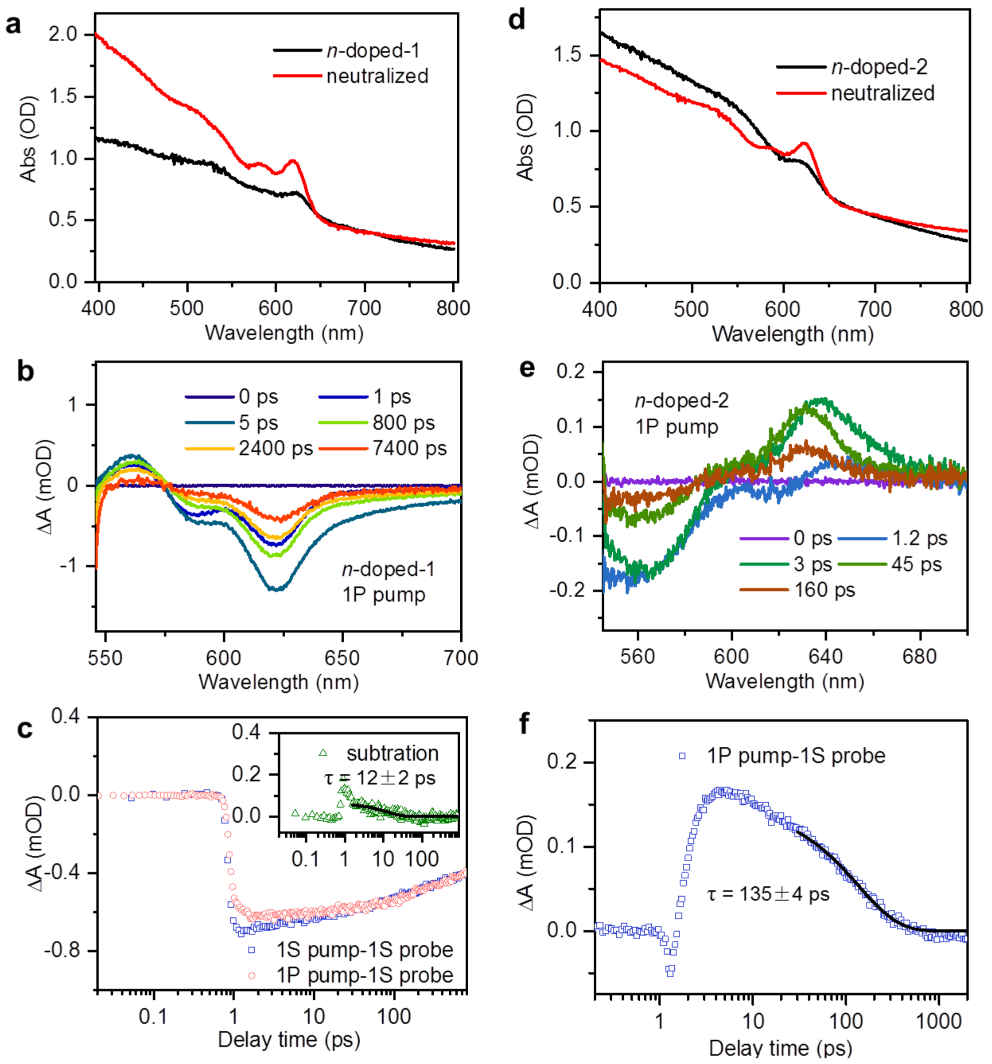

Supplementary Figure 13. Hot electron lifetime *n*-doped QD films. (a) Absorption spectra of a lightly-doped QD film (black) and its corresponding neutralized film (red).The optical scattering prohibits us from determining the nominal number of doped band-edge electrons in the film. Nonetheless, we can still semi-quantitatively control the number of doped electrons by controlling the amount of reducing reagent used. (b) TA spectra of the lightly-doped QD film at indicated delays under 1P excitation. (c) TA kinetics of the lightly-doped QD film probed at the 1S bleach feature under 1S (blue squares) and 1P (red circles) pump conditions. Inset is the difference between the two kinetics (green triangles) and its fit (black solid line). (d) Absorption spectra of a heavily-doped QD film (black) and its corresponding neutralized film (red). (e) TA spectra of the heavily-doped QD film at indicated delays under 1P excitation. (f) TA kinetics of the heavily-doped QD film probed at the 1S absorption feature under 1P pump (blue squares). The black solid line is a fit to the decay kinetics.

Supplementary Reference:

1 Ding, T., Liang, G., Wang, J. & Wu, K. Carrier-doping as a tool to probe the electronic structure and multi-carrier recombination dynamics in heterostructured colloidal nanocrystals. *Chem. Sci.* **9**, 7253-7260 (2018).
